# Postoperative *Staphylococcus aureus* Infections in Medicare Beneficiaries

**DOI:** 10.1371/journal.pone.0110133

**Published:** 2014-11-12

**Authors:** Moaven Razavi, Donald S. Shepard, Jose A. Suaya, William B. Stason

**Affiliations:** 1 Schneider Institutes for Health Policy, Heller School for Social Policy and Management, Brandeis University, Waltham, Massachusetts, United States of America; 2 GlaxoSmithKline, Philadelphia, Pennsylvania, United States of America; Columbia University, College of Physicians and Surgeons, United States of America

## Abstract

*Staphylococcus aureus (S. aureus)* infections are important because of their increasing frequency, resistance to antibiotics, and high associated rates of disabilities and deaths. We examined the incidence and correlates of *S. aureus* infections following 219,958 major surgical procedures in a 5% random sample of fee-for-service Medicare beneficiaries from 2004–2007. Of these surgical patients, 0.3% had *S. aureus* infections during the hospitalizations when index surgical procedures were performed; and 1.7% and 2.3%, respectively, were hospitalized with infections within 60 days or 180 days following admissions for index surgeries. *S. aureus* infections occurred within 180 days in 1.9% of patients following coronary artery bypass graft surgery, 2.3% following hip surgery, and 5.9% following gastric or esophageal surgery. Of patients first hospitalized with any major infection reported during the first 180 days after index surgery, 15% of infections were due to *S. aureus*, 18% to other documented organisms, and no specific organism was reported on claim forms in 67%. Patient-level predictors of *S. aureus* infections included transfer from skilled nursing facilities or chronic hospitals and comorbid conditions (e.g., diabetes, congestive heart failure, chronic obstructive pulmonary disease, and chronic renal disease). In a logarithmic regression, elective index admissions with *S. aureus* infection stayed 130% longer than comparable patients without that infection. Within 180 days of the index surgery, 23.9% of patients with *S. aureus* infection and 10.6% of patients without this infection had died. In a multivariate logistic regression of death within 180 days of admission for the index surgery with adjustment for demographics, co-morbidities, and other risks, *S. aureus* was associated with a 42% excess risk of death. Due to incomplete documentation of organisms in Medicare claims, these statistics may underestimate the magnitude of *S. aureus* infection. Nevertheless, this study generated a higher rate of *S. aureus* infections than previous studies.

## Introduction

Postoperative infections convey substantially increased clinical risks and increases in health care costs. Infections by *Staphylococcus aureus (S. aureus)*, other gram positive organisms, including *Clostridium difficile*, gram-negative organisms including pseudomonas, *Escherichia. coli*, enterococci, and fungal infections are important because of their increasing frequencies, resistance to antibiotics, and associated deaths and disability [1]. Infections due to *S. aureus* are of particular concern in the face of increasing methicillin resistance (MRSA). A retrospective study of surgical patients based on medical records found 0.47 invasive *S. aureus* infections per 100 procedures, of which 51% were due to MRSA [2]. In that study, cardiothoracic procedures had the highest infection rate (0.79 per 100), followed by 0.62 per 100 for neurosurgical procedures, and 0.37 per 100 for orthopedic procedures. Among 133,450 *S. aureus* infections isolated from 1998 to 2009 from medical and surgical patients in an integrated health plan, 40% were MRSA [3].

Efforts to reduce the frequency of *S. aureus* infections following surgical procedures have focused both on prevention and treatment. Preventive measures aim to reduce nosocomial infections transmitted to patients by hospital workers or hospital facilities [4], [5] or on identifying and treating surgical patients who are carriers of MRSA organisms when they enter the hospital [4], [6], [7], [8], [9]. Use of the multicomponent MRSA bundle (nasal screening, contact isolation, hand hygiene, leadership, and monitoring) has been very effective in reducing the frequency of postoperative infections [10]. We focused our study on cardiovascular, orthopedic and gastro-intestinal surgery due to a combination of their high frequency in Medicare beneficiaries and the risks of *were 15.0%* infection in the literature.

Infections related to cardiothoracic surgery are mainly wound infections, septicemia, endocarditis, and pneumonia [11]. Patients who have surgery on the ascending aorta and require 48 hours or more mechanical ventilation following the procedure or require reoperation and heart transplant recipients are at especially high risk [12]. Among orthopedic surgery procedures, knee or hip replacement or arthroplasty are accompanied by especially high risks of post-operative infections, with *S. aureus* being the most frequent organism cultured [13], [14]. A large questionnaire study found that infections following orthopedic surgery are usually not detected until after discharge from the surgical admission, often at a subsequent hospital admission [15].

To our knowledge, previous studies of postoperative infections caused by *S. aureus*, based on medical records, were limited to clinical populations in a few institutions and a few weeks of follow up. Our study uses claims data for a large and nationally representative sample of Medicare beneficiaries. To avoid missing delayed effects, we examine the occurrence of *S. aureus* infections for up to 180 days following common cardiovascular (CV), orthopedic, or gastrointestinal (GI) surgical procedures among Medicare beneficiaries. We also examine the effects of S. *aureus* infection on length of stay and mortality.

## Methods

### Ethics statement

The study was approved by the Brandeis University Committee for the Protection of Human Studies in Research. As the study was based entirely on existing data without patient names or identifying numbers, no patient consent was required.

### Data source

This study used Medicare entitlement data and Part A claims data (primarily inpatient hospital care) from the random 5% sample of beneficiaries in Medicare's chronic condition warehouse (CCW). The claims for such persons in the CCW as of Jan. 1, 2004 plus new entrants to the CCW through 2007 were pooled to construct the study's analytical file [16].

### Index surgeries

Three main categories of surgery were studied: CV, orthopedic and GI surgeries. CV surgeries were classified into coronary artery bypass graft (CABG), percutaneous coronary interventions (PCI), and other. PCI was included because it is the most frequent cardiovascular procedure, was usually done as an inpatient procedure in Medicare beneficiaries, and is often an alternative to CABG [17]. Orthopedic surgeries were grouped into hip, knee, or other. GI surgeries were classified into gastric, laparotomy, or other. Surgical procedures were identified by specific International Classification of Diseases (ICD) inpatient procedure codes listed in **Table S1**. Codes were selected based on review of coding manuals, prior literature, and the medical knowledge of the two physician authors (WS, JS).

### Study design

The design was a retrospective cohort study consisting of Medicare fee-for-service patients in the 5% sample who underwent one or more of the specified types of surgery during an acute hospital admission. For the patient to be in our cohort, the procedure needed to be performed between July 1, 2004 and June 30, 2007 and be recorded in the inpatient claim paid by Medicare. The patient's first such admission was termed the index hospitalization, and each such index hospitalization was a unit of observation.

To provide context for *S. aureus* infections, we examined the occurrence of the first hospitalization with a “specified” infectious diagnosis within the follow up period. These specified infections, identified by the ICD-9 discharge codes listed in **Table S2** (ICD 9 codes 001–139), represented 15.0% of potential index admissions where the patient had been hospitalized in the preceding 6 months with any specified infection.

The follow up period began with the date of the index surgery and continued for 180 days thereafter. We selected this period to include infections which progress slowly. Infections that occurred during the index hospitalization were labeled as day 0 because the discharge diagnosis from this hospitalization was the first documentation of such infections in the claims data.

Post-operative *S. aureus* infections were those for which this first specified infection was related to *S. aureus*. These were identified primarily by the occurrence of any of the following *S. aureus*-specific ICD-9 codes among the hospital discharge diagnoses: septicemia (0.38.1x), meningitis (3203), infections of the circulatory system (42292), pneumonia (482.4x) and other infections (041.1x). (For the complete list, see starred items in **Table S2**.)

### Post-operative *S. aureus* infection rates

We evaluated rates of first *S. aureus* infections within 180 days following the index surgery for each category and type of surgery of interest. *S. aureus* infections were then characterized by (1) the category and type of index procedure as described above; (2) the type of infection according to the hierarchy we developed corresponding to typical severity: invasive (septicemia, meningitis and infections of the circulatory system codes), pneumonia, or other; and (3) the timing of the first hospitalization with this infection diagnosis (same admission as the index surgical procedure or a subsequent hospital admission). As a supplemental analysis, we also counted all hospitalizations with a *S. aureus* infection during the 180-day follow-up period, whether or not they were the first such hospitalization.

### Predictors of post-operative *S. aureus* infection

Negative binomial regression was used to predict the risk of first *S. aureus* infections within 180 days following the index surgery as incidence rate ratios (IRR). This approach adjusts for each patient's period of exposure to first infection. Independent regressions were performed for major types of surgery. For an additional regression including all surgeries, patients were classified by the category of the index surgery: CV, orthopedic, or GI. We used key case mix severity measures to identify potential predictors of *S. aureus* infections following surgeries. Patient demographics consisted of sex, race (white or non-white), and age group (by decade). Medicare eligibility was stratified as due to disability, end stage renal disease (ESRD), or age. Two main characteristics of the index hospitalization for surgery were used: source of the admission (previous institution vs. community) and whether or not the index hospitalization was elective or non-elective (including unknown). Five comorbid conditions were included: congestive heart failure, ischemic heart disease, chronic obstructive pulmonary disease (COPD), chronic renal disease, and solid cancers. Finally, indicators for four census regions were also included in the model (Mid-West, South, West, and the combination of Northeast and Mid-Atlantic). Logistic regression was used to validate the findings.

### Predictors of length of stay

To examine the impact of *S. aureus* infection on hospital stay we focused on the index admission. Despite our exclusion period, an index patient could have developed an infection immediately prior to the index admission. As surgeons generally do not perform elective procedures on patients with an infection, we performed an alternative analysis limited to elective procedures. As lengths of stay are skewed, we used the logarithmic transformation.

### Predictors of post-operative death

Logistic regression was used to predict the risk of death within 180 days following the index surgery. The same predictors were used as those when predicting infection with the addition of *S. aureus* infection, including focusing exclusively on elective admissions.

### Sensitivity analyses

To control for possible confounding from infections during the follow-up period arising from causes unrelated to the index surgery, we performed sensitivity analyses with 30-day and 60-day follow up periods. A shorter period of follow-up reduces the risk of included unrelated infections, but may miss procedure-related infections that develop slowly. As a sensitivity analyses,

## Results

### Study Population

The CCW for the years 2004 through 2007 contains about 2.5 million Medicare beneficiaries. Due to deaths and new entrants, the size slightly exceeds 5% of the 47 million Medicare beneficiaries in 2007 [Chronic Conditions Data Warehouse, www.ccwdata.org, accessed 4/11/14]. Of these, 257,240 Medicare patients underwent a CV, GI, or orthopedic procedure of interest during the three-year study period. Following exclusions of patients who had not been randomly chosen (i.e., lacked the 5% random flag), belonged to a Medicare Advantage plan or Health Maintenance Organization during any part of the study period, or lacked follow-up date, 219,958 beneficiaries were included in the analysis. **Table 1** shows their characteristics.

**Table 1:** Descriptive characteristics and outcomes of study population of Medicare beneficiaries with selected index surgeries.

	Pts. with index surgery		Pts.w/index surgery and SA infection		Deaths in patients with index surgery	
Characteristic	Number	% of total	Number	% of pts. w/index surgery	Number	% of pts. w/index surgery
Sex						
Males	98,194	45%	2,467	2.5%	11,259	11.5%
Females	121,764	55%	2,610	2.1%	12,761	10.5%
Race						
White	194,262	88%	4,215	2.2%	20,022	10.3%
Non-White	25,696	12%	862	3.4%	3,998	15.6%
Age						
64andyounger	24,694	11%	892	3.6%	1,755	7.1%
65–74years	82,137	37%	1,456	1.8%	5,200	6.3%
75–84years	80,329	37%	1,827	2.3%	9,389	11.7%
85+ years	32,798	15%	902	2.8%	7,676	23.4%
Admission source						
Home	203,709	93%	4,533	2.2%	21,325	10.5%
Acute health care facility	14,527	7%	452	3.1%	2,188	15.1%
SNF	1,210	1%	74	6.1%	443	36.6%
Missing	512	0%	18	3.5%	64	12.5%
Medicare eligibility						
Disability or ESRD	25,364	12%	917	3.6%	1,873	7.4%
Age 65+, no disability or ESRD	194,594	88%	4,160	2.1%	22,147	11.4%
Region						
Northeast and Mid-Atlantic	38,840	18%	916	2.4%	4,530	11.7%
Midwest	60,542	28%	1,222	2.0%	6,149	10.2%
South	88,824	40%	2,136	2.4%	10,127	11.4%
West	31,616	14%	801	2.5%	3,198	10.1%
International and other	136	0%	2	1.5%	16	11.8%
Type of admission during index surgery						
Elective	105,821	48%	1,760	1.7%	4,668	4.4%
Non-elective	114,137	52%	3,317	2.9%	19,352	17.0%
Comorbidities present at index admission+						
Diabetes	84,154	38%	2,632	3.1%	10817	12.9%
Congestive heart failure	94,348	43%	3,295	3.5%	17003	18.0%
Ischemic heart disease	151,290	69%	3,702	2.4%	18217	12.0%
COPD	69,200	31%	2,441	3.5%	11973	17.3%
Chronic renal disease	56,009	25%	2,463	4.4%	12780	22.8%
Solid cancer	36,209	16%	875	2.4%	5563	15.4%
Type of index surgery						
Cardiovascular surgery	71,404	32%	966	1.4%	4,611	6.5%
Orthopedic	93,907	43%	2,014	2.1%	6,370	6.8%
Gastrointestinal	54,647	25%	2,097	3.8%	13,039	23.9%
Total	219,958	100%	5,077	2.3%	24,020	10.9%

Note: SA denotes *Staphylococcus aureus*, SNF denotes skilled nursing facility, ERSD denotes end stage renal disease, COPD denotes chronic obstructive pulmonary disease, Pts. denotes patients.

+ Note that the co-morbodities are not mutually exclusive dummy variables. A patient can have multiple comorbities so the sums of the groups exceed the total population.

Within our analysis population, 71,404 patients had undergone a cardiac procedure, 54,647 a GI procedure, and 93,907 an orthopedic index procedure. Of our analysis population, 33,868 (15.4%) had a hospital-based diagnosis for a major infection (i.e., one listed in **Table S2**) within 180 days following surgery. Of these patients, 66% had a single procedure and one infection, 23% had a single procedure and multiple infections, and the remaining 11% had multiple surgical procedures and one or more infections during the follow-up period. Hence, about one-third of patients had repeated surgeries or multiple infection admissions.

Rates of *S. aureus* infections treated during hospital admissions in the 60 and 180 days following the index surgical procedure are shown in **Table 2**. Overall, *S. aureus* infection was identified during or following 5,077 surgeries. Specifically, *S. aureus* infection rates were 1.7% within 60 days and 2.3% within 180 days from surgery, representing 15.0% of the major infections.

**Table 2:** Types and frequencies of first *S. aureus* infections following index surgeries.

	Pts. with index surgery	% pts.w/infection, 60 days	% pts.w/infection, 180 days	Net% w/infection, 180 days	% distribution by type			
Type of surgery					Num-ber	In-vasive	Pneu-monia	Other
Cardiovascular surgeries	71,404	0.9%	1.4%	0.8%	966	35%	18%	47%
CABG	15,356	1.4%	1.9%	1.6%	287	29%	13%	58%
PCI	43,814	0.6%	0.9%	0.4%	412	42%	18%	40%
Other	12,234	1.5%	2.2%	1.0%	267	31%	22%	47%
Orthopedic surgeries	93,907	1.6%	2.1%	1.6%	2,014	20%	7%	73%
Hip	26,386	1.7%	2.3%	2.0%	594	20%	9%	71%
Knee	35,114	0.7%	1.0%	0.5%	359	17%	5%	78%
Other	32,407	2.4%	3.3%	2.4%	1,061	21%	7%	72%
Gastro-intestinal surgeries	54,647	2.9%	3.8%	2.8%	2,097	34%	25%	42%
Gastric & esophogeal	20,271	4.5%	5.9%	4.4%	1,193	34%	32%	34%
Laparotomy	9,005	2.0%	2.8%	2.0%	256	24%	12%	64%
Other	25,371	1.9%	2.6%	1.7%	648	37%	16%	47%
All types	219,958	1.7%	2.3%	1.6%	5,077	28%	16%	55%

Note: *S. aureus* denotes *Staphylococcus aureus*; pts. denotes patients; CABG denotes coronary artery bypass graft; PCI denotes percutaneous coronary intervention, w/denotes with. First *S. aureus* infection refers to first hospitalization with or following index surgery with an *S. aureus* discharge diagnosis code. The % distribution refers to patients with *S. aureus* infection within 180 days of index surgery. Type of infection is based on the hierarchy: invasive (septicemia, meningitis, and infections of the circulatory system, ICD-9 codes 038xx, 3203, 42292), pneumonia (codes 4824x), other (codes 0411x).

*S. aureus* infection rates varied by category of surgery, being 1.4% following cardiac procedures, 2.1% after orthopedic procedures, and 3.8% after GI procedures. Infection rates also varied by type of surgery: 0.9% of patients after percutaneous coronary intervention (PCI), 1.9% after CABG surgery, 1.0% after knee procedures, 2.3% after hip procedures, 5.9% after gastric or esophageal procedures, 2.8% after laparotomies, and 2.3% overall. *S. aureus* was the first listed infection in 80% of these cases with a *S. aureus* infection, and a concurrent infection in the remainder (table not shown). Our supplemental analysis found that 1,487 *S. aureus* infections occurred during later hospitalizations, in addition to the 5,077 *S. aureus* infections on the initial hospitalization already included in our study. Inclusion of these infections would have increased the incidence of *S. aureus* infections to 3.0% instead of 2.3%.

### *S. aureus* Infection Rates by Site of Infection

Invasive infections, including septicemia, bacterial endocarditis, and meningitis were the most frequent clinical presentations for infections caused by *S. aureus* (28% of all *S. aureus* infections), followed by pneumonia in 16% of cases, and miscellaneous other types of infection in 55% (see **Table 2**). Infection types are important considerations. Our study found that 35% of infections following cardiac or GI procedures were “invasive”—mostly reflecting septicemia—compared to 20% following orthopedic procedures. The share for pneumonia ranged from 7% following orthopedic procedures to 18% after cardiac procedures and 25% following or GI procedures. More than half (55%) of infections involved other sites.

### Time to First *S. aureus* Infection

Overall, slightly more than half of first *S. aureus* infections occurred within 30 days of the index surgery. Cumulatively, 14% of first infections occurred during the index admission, 56% within 30 days, and 73% within 60 days. Among patients with cardiac procedures, 13% of the first *S. aureus* infections occurred during the index admission, 49% within 30 days, and 67% within 60 days. Among patients with GI procedures, these shares were 12%, 59%, and 74%, respectively. Among beneficiaries with orthopedic surgery, these shares were 16%, 57%, and 74%, respectively. Thus, the share of infections during the index hospitalization is greatest for orthopedic surgeries, though the differences are small.

### Patient-Level Predictors of Post-Operative *S. aureus* Infection

For CABG surgery, significant positive associations were identified in patients who had become eligible for Medicare because of an underlying disability and those who had comorbidities including diabetes mellitus, congestive heart failure, chronic obstructive pulmonary disease, and chronic renal disease (**Table 3**). In most cases, these factors also predicted post-operative infections for the other types of surgical procedure. In addition, higher risks of *S. aureus* infections were found following PCI, gastric or knee surgery in patients referred from other acute or chronic care facilities; for patients following gastric procedures, those who lived in the southern and western regions of the US (vs. the East or mid-Atlantic regions). The validation results using multivariate logistic regression, shown in **Table S3**, were generally similar. In addition, sensitivity analysis of incidence rate ratios for 30 days found generally similar results in significance and signs of coefficients to those with 180 days (data not shown). The one exception was for the variable race non-white, which lost significance in the 30-day analysis. The pseudo R-squared was higher for 180-day analysis (0.127) than the 30-day one (0.100).

**Table 3:** Adjusted predictors of *S. aureus* infection within 180-days following surgery by type of index surgery based on incidence rate ratio (IRR).

Predictor	Cardiovascular				Gastro-intestinal				Orthopedic				All	
	CABG		PCI		Gastric		Laparo-tomy		Hip		Knee			
Female vs. male	1.28	*	1.05		0.71	*	0.74	*	0.76	*	0.85		0.78	*
Non-white vs. white	0.72		0.96		1.11		1.06		1.19		0.97		1.09	*
Age 75–84 vs. <75	1.08		1.06		1.03		0.89		1.11		1.06		1.05	
Age 85+ vs. <75	1.33		0.78		1.01		1.08		1.15		1.17		1.07	
Source: SNF	0.00		1.50		1.59	*	1.94		0.65		5.76	*	1.90	*
Source: Acute or chronic hospitals or rehab vs. community	1.07		1.34	*	1.78	*	1.59		1.37		4.09	*	1.58	*
Source Missing	2.31		1.78		1.45		0.00		1.62		0.00		1.62	*
Disability and ESRD vs. age eligibility	1.50	*	1.81	*	1.00		1.51	*	2.47	*	2.59	*	1.57	*
Region MidWest vs. NEast and MidAtlan	0.93		0.97		1.18		0.87		0.81		0.91		0.95	
Region South vs. NEast and MidAtlan	0.93		0.88		1.28	*	1.01		1.20		0.97		1.07	
Region West vs NEast and MidAtlan	1.03		0.97		1.61	*	0.89		1.18		1.19		1.18	*
Elective vs. non-elective admission for index surgery	0.89		0.66	*	0.53	*	1.01		0.53	*	0.46	*	0.68	*
Diabetes (yes vs. no)	1.61	*	1.36	*	1.18	*	1.20		1.26	*	1.37	*	1.27	*
Congestive heart failure (yes vs. no)	2.67	*	3.06	*	1.62	*	1.86	*	1.65	*	1.82	*	2.07	*
Ischemic heart disease (yes vs. no)	n.a.		n.a.		0.97		0.93		1.07		1.33	*	0.98	
COPD (yes vs. no)	1.50	*	1.75	*	1.65	*	1.72	*	1.60	*	1.41	*	1.62	*
Chronic renal disease (yes vs. no)	2.79	*	3.33	*	1.60	*	2.49	*	1.87	*	2.49	*	2.29	*
Solid cancer (yes vs. no)	1.03		1.23		0.88		1.30		0.94		1.18		0.85	*
Gastro-intestinal vs. cardiovascular surgery	n.a.		n.a.		n.a.		n.a.		n.a.		n.a.		4.54	*
Orthopedic vs. cardiovascular surgery	n.a.		n.a.		n.a.		n.a.		n.a.		n.a.		2.82	*
McFadden's pseudo R-squared	0.11		0.15		0.07		0.07		0.08		0.09		0.13	
Number of patients (000)	15		44		20		9		26		35		220	
Cross-tabulated non-zero cells	2796		4308		5475		3252		4083		3123		20,158	

Note: *S. aureus* denotes *Staphylococcus aureus*. *S. aureus* infection refers to first hospitalization coinciding or following surgery of interest with discharge diagnoses including any ICD-9 code specific for infection due to *S. aureus*. Rehab denotes rehabilitation; SNF denotes skilled nursing facility; ESRD denotes end-stage renal disease; NEast denotes northeast; MidAtlan denotes mid-Atlantic; COPD denotes chronic obstructive pulmonary disease; n.a. denotes not applicable, vs. denotes versus. IRR>1 denotes factor associated with higher risk; IRR<1 denotes lower risk. Each IRR regression was based on all cross-tabulated non-zero cells. Based on log likelihood ratio Chi-squared, the overall regression tests for all six procedures plus the pooled group were highly significant (p<0.0001). *denotes p<0.05.

### Post-Surgical Mortality

Deaths within 180 days following surgical procedures occurred in 23.9% of all patients with *S. aureus* infections and 10.6% of patients without a post-operative *S. aureus* infection (**Table 4**). Hence, *S. aureus* infection was associated with an unadjusted 2.3-fold increased risk of death. Case-fatality rates within 180 days in patients with *S. aureus* infections ranged from 4.7% following knee procedures to 42.0% following gastric or esophageal surgical procedures, compared with 0.7% and 38.2%, respectively, in the absence of *S. aureus* infection. For other surgical procedures, case-fatality rates with *S. aureus* infections were 15.0% vs. 6.3% without *S. aureus* infections for CABG, 16.5% vs. 4.9% for PCI, 20.0% vs. 8.1% for hip surgery, and 19.5% vs. 14.0% for laparotomy. Hence, *S. aureus* infections were associated with substantially higher procedure-specific crude mortality rates. In addition, the wide range in mortality rates among procedures in the absence of infection suggests that case mix variation is substantial.

**Table 4:** Unadjusted mortality rates within 180 days following index surgery by *S. aureus* infection status.

Type of surgery	With *S. aureus* infection				Without *S. aureus* infection				Mortality	
	Patients	CFR ± SE			Patients	CFR ± SE			risk ratio	
Cardiac surgeries	966	18.4%	±	1.2%	70,438	6.3%	±	0.1%	2.93	**
CABG	287	15.0%	±	2.1%	15,069	6.3%	±	0.2%	2.38	**
PCI	412	16.5%	±	1.8%	43,402	4.9%	±	0.1%	3.40	**
Other	267	25.1%	±	2.7%	11,967	11.5%	±	0.3%	2.18	**
Orthopedic surgeries	2,014	16.8%	±	0.8%	91,893	6.6%	±	0.1%	2.56	**
Hip	594	20.0%	±	1.6%	25,792	8.1%	±	0.2%	2.46	**
Knee	359	4.7%	±	1.1%	34,755	0.7%	±	0.0%	6.86	**
Other	1,061	19.0%	±	1.2%	31,346	11.8%	±	0.2%	1.62	**
Gastro-intestinal surgeries	2,097	33.3%	±	1.0%	52,550	23.5%	±	0.2%	1.42	**
Gastric & esophogeal	1,193	42.0%	±	1.4%	19,078	38.2%	±	0.4%	1.10	**
Laparotomy	256	19.5%	±	2.5%	8,749	14.0%	±	0.4%	1.40	*
Other	648	22.7%	±	1.6%	24,723	15.5%	±	0.2%	1.46	**
All types	5,077	23.9%	±	0.6%	214,881	10.6%	±	0.1%	2.25	**

Note: *S. aureus (SA)* infection refers to first hospitalization coinciding with or following the index surgical procedure with a discharge diagnosis of SA. These diagnoses include ICD-9 codes with SA for: septicemia, meningitis, infections of the circulatory system, pneumonia, and others (ICD-9 codes 038xx, 3203, 42292, 4824x, and 0411x), respectively. CFR ± SE denotes case fatality rate with its standard error. *denotes p<0.05. **denotes p<0.01.

The length of stay of the 686 patients (±standard error of the mean) with *S. aureus* infection on their index admission was 14.8±0.6 days. For the remaining 219,272 patients, the length averaged 7.1±0.02 days. The multi-variable log analysis of LOS showed that *S. aureus* was associated with highly significant factor of 3.10 for all surgeries and 2.39 for elective admissions (**Table 5**). These regressions also showed, as expected, that older age, prior stay in a SNF, and co-morbidities were associated with longer lengths of stay.

**Table 5:** Adjusted predictors of outcomes following index surgeries.

	Exponentiated reg. coefficients on LOS of index admission				Odds ratio for death within 180 days			
Predictor	All Surgeries		Elective Only		All Surgeries		Elective Only	
Post-operative *S. aureus* infection (1 = yes vs. 0 = no)&	3.10	****	2.39	****	1.42	****	2.05	****
Female (1) vs. male (0)	1.42	****	1.53	****	0.82	****	0.88	****
Non-white (1) vs. white (0)	0.99	**	0.99	NS	1.10	****	1.12	*
Age 75–84 (1) vs. 0–74 yrs. (0)	1.09	****	1.06	****	1.52	****	1.57	****
Age 85+ (1) vs. 0–74 yrs. (0)	1.06	****	1.06	****	2.72	****	2.90	****
Index hospitalization source: SNF (1) vs. community (0)	1.07	****	1.08	****	1.84	****	3.32	****
Source: Acute, or chronic hosp, or rehab (1) vs. community (0)	1.02	NS	1.22	***	1.37	****	2.45	****
Source: Missing (1) vs. community (0)	1.20	****	1.58	****	1.20	NS	1.80	*
Disability and ESRD (1) vs. age eligibility (0)	1.02	NS	1.06	NS	0.83	****	0.81	***
Region MidWest (1) vs. NEast and MidAtlantic (0)	0.93	****	0.89	****	1.05	*	1.27	****
Region South (1) vs. NEast and MidAtlantic (0)	0.95	****	0.96	****	1.15	****	1.44	****
Region West (1) vs NEast and MidAtlantic (0)	0.98	****	1.00	NS	1.04	NS	1.24	***
RegionX: International (1) vs. NEast and MidAtlantic (0)	0.95	****	0.96	****	1.14	NS	0.55	NS
Elective(1) vs. non-elective admission for index surgery (0)	1.11	NS	1.08	NS	0.37	****	n.a.	n.a.
Diabetes (1 = yes vs.0 = no)	0.75	****	0.00	n.a.	0.92	****	0.95	NS
Congestive heart failure (1 = yes vs. 0 = no)	1.03	****	1.01	NS	2.08	****	2.23	****
Ischemic heart disease (1 = yes vs. 0 = no)	1.33	****	1.27	****	0.95	*	1.00	NS
COPD (1 = yes vs. 0 = no)	0.97	****	0.97	****	1.54	****	1.77	****
Chronic renal disease (1 = yes vs. 0 = no)	1.12	****	1.11	****	2.48	****	3.02	****
Solid cancer (1 = yes vs. 0 = no)	1.30	****	1.27	****	1.12	****	1.06	
Gastro-intestinal (1) vs. cardiovascular surgery (0)	1.04	****	1.04	****	4.48	****	3.66	****
Orthopedic (1) vs. cardiovascular surgery (0)	2.31	****	2.16	****	1.35	****	0.59	****

Note. *S. aureus* denotes *Staphylococcus aureus*. SNF denotes skilled nursing facility; Rehab denotes rehabilitation; ESRD denotes end-stage renal disease; NEast denotes northeast; MidAtlan denotes mid-Atlantic; COPD denotes chronic obstructive pulmonary disease, vs. denotes versus; c-statistic is 0.83; *denotes p<0.05; **denotes p<.01, ***denotes p<.001, ****denotes p<.0001, NS denotes not significant, n.a. denotes not applicable. Adjusted odds ratios were obtained from binary logistic multivariate regression performed on 219,958 patients with an index surgery, 5,077 of them with at least one hospitalization associated with *S. aureus* infection within 180 days from index surgery. A total of 24,020 patients died within 180-days from index surgery. SA infection refers to index admission only for length of stay, and any admission within 180 days of index admission in models for death.

The logistic regression analysis found that mortality was significantly higher in patients with *S. aureus* infection, male gender, older age, referral for the surgical procedure from a skilled nursing facility (SNF) or chronic hospital, surgery performed in the South or Midwest, non-elective admissions, comorbidities including congestive heart failure, chronic renal disease, or COPD, and gastrointestinal or orthopedic surgery. The strongest associations based on odds ratios (ORs) in descending order are GI versus CV surgery (OR 4.5), age 85 or above (OR 2.7) versus age 65–74, presence of chronic renal disease (OR 2.5) or congestive heart failure (OR 2.1). Elective surgery, compared with emergent surgery, was strongly protective (OR 0.4). *S. aureus* infection was predictive of higher mortality with an OR of 1.4.

## Discussion

Overall, 2.3% of these surgical patients had a documented *S. aureus* infection. Infection rates were highest following GI procedures, followed by cardiac and orthopedic procedures, and in patients with emergent versus elective surgery.

*S. aureus* was associated with more than doubling the length of stay of elective admissions after controlling for other predictors. Deaths within 180 days following surgery occurred in 23.9% of patients with a *S. aureus* infection but only 10.6% of those without a *S. aureus* infection. These results reinforce the importance of infection control measures, particularly in patients at higher risk. The strongest predictors of post-operative deaths were transfer from a SNF or chronic hospital, non-elective admissions, and chronic disease comorbidities.

Claims data cannot completely determine the cause of each post-surgical infection. It could occur due to exposure during the index surgery, other exposures during that hospitalization (e.g., central line or ventilator), related subsequent medical care, increased proclivity to infection as a result of the index surgery, or completely unrelated factors. Our sensitivity analyses with 30-day and 60-day follow-up periods from the index surgery obtained probabilities of *S. aureus* of 1.3% and 1.7%, respectively. As these are 56% and 73% of the 180-day rate, respectively, even our most conservative sensitivity analysis (30 days) links the majority of infections closely in time to the index surgery. These patterns are also consistent with the median times to identifying surgical site and blood stream infections (19 and 18 days, respectively) in a previous study [2]. Furthermore, the distributions among types infections were similar the 60-day and 180-day follow up period, suggesting that our more comprehensive period of 180 days did not introduce distortions.

This study presents several advances over previous research. First, the present sample size (N = 219,958) is more than twice the size of the largest previous study. Second, the present study is based on a national, random sample of Medicare fee-for-service claims. Third, our study examined a variety of sites of infection, not just invasive surgical site and bloodstream infections. Fourth, this study provides substantial detail on elderly patients, since almost all of the patients were over age 65. Fifth, the follow-up to 180 days allowed us to include infections that took months to become apparent and diagnose. Sixth, the study had almost no losses to follow up as the Medicare program tracks mortality and virtually all hospitalizations of fee-for-service beneficiaries. These design features expanded evidence about *S. aureus* infection, contributing to a cumulative incidence in our study of 5 times the rate of 0.47% from the major previous study were 15.0% [2].

The present study's large sample size allowed two analyses that were not included in previous work. The first was a statistical analysis of predictors of infection using multivariable logistical regression and IRRs. The most important predictors of risk of *S. aureus* infection were the type of surgery (gastrointestinal and orthopedic, compared to cardiovascular), Medicare eligibility based on disability or ESRD, the index surgical procedure being non-elective (compared to elective), and the presence of at least one specified co-morbidity, including diabetes, congestive heart failure, or chronic obstructive pulmonary disease.

The second sub-analysis concerned predictors of mortality within 180 days of the index discharge. In general, these predictors tended to be comparable to those for *S. aureus* infection. For example, a diagnosis of congestive heart failure had an IRR for infection of 2.07 and an OR for death of 2.08, both numbers being significantly different from 1.00 (the value for no effect). The major difference between the two analyses is that the mortality regression included *S. aureus* infection as a covariate, finding a statistically significant OR of 1.42, corresponding to an additional 42% risk. A few variables (e.g. diabetes) changed sign between the two analyses. Diabetes was associated with a 27% excess risk of infection, but a reduced 8% risk of death, given infection. This contrast indicates that diabetes appears to operate through a proclivity to infection, whereas congestive heart failure both increases the risk of infection as well as the risk that the infection proves fatal.

Limitations of our study need to be acknowledged. For three reasons, our study probably under-estimated the true rate of post-operative *S. aureus* infections. First, the analysis depended importantly on the reliability and completeness of Medicare claims data in identifying and characterizing episodes of infectious disease that occurred during hospital admissions. These claims specified the causative organism for only a third of all specified infections. This pattern reflects clinical practices to initiate treatment without bacteriologic documentation or the failure to include these data in hospital discharge summaries, or omission or truncation of diagnosis details in Medicare claims. As identification of the infective organism (often shown as the fourth digit of an ICD-9 diagnosis code) generally does not affect the Diagnosis Related Group (DRG), it generally does not affect Medicare payment for a hospitalization. However, due to the risk of MRSA, testing for and documentation of *S. aureus* infection is likely more complete than that for other organisms. Second, our main analysis examined only the first hospitalization for each patient who had an infectious disease listed as a discharge diagnosis (e.g. truncating ICD-9 codes to three digits). Our supplemental analysis showed that the inclusion of later hospitalizations would raise the rate of *S. aureus* infection by about 30%. Third, our analysis did not include complications of surgery listed under ICD-9 code 998.5, for surgical infections. If coders had entered only that code and failed to follow the instruction to include a specific diagnostic code for the exact infection, we could have missed some *S. aureus* infections.

Finally, our analysis examined only the chronological relationship between the index surgery and infection, but could not examine the etiology. It is possible that some chronologically linked infections were not caused by the surgery. As noted, 11% of *S. aureus* infections were reported at the discharge containing the index surgery. Discharge diagnoses do not allow us to determine whether or not this infection was present when the patient was admitted, although it is unlikely that surgeons would have performed elective surgery on a patient known to have this infection. Some of the infections 61 to 180 days following the index surgery, which represented 27% of post-operative *S. aureus* infections, also may not have been related to the index surgery.

In conclusion, the 2.3% rate of *S. aureus* post-operative infections in this claims-based study is 4.9 times the rate in the largest previous study [2]. The main reasons for the higher rate in this study are an older population (mean age 75 years vs. 57 years) and a wider range of infection types (all vs. invasive and wound site only). After controlling for other factors, *S. aureus* infection was associated with doubling the stay of index hospitalization and a 42% excess risk of death within 180 days of the index surgery. These findings suggest that lowering of the risk *S. aureus* infection would reduce hospital readmission, hospital days, and mortality in Medicare beneficiaries.

## Supporting Information

Table S1 **ICD-9 procedure codes for surgical procedures.**
(DOCX)

Table S2 **ICD-9 diagnosis codes for infections.**
(DOCX)

Table S3 **Adjusted predictors of** ***S. aureus*** **infection within 180 days following surgery by type of surgery based on odds ratios (OR).**
(DOCX)
