# Supplementary material for: Postoperative Staphylococcus aureus Infections in Medicare Beneficiaries
Source: PLoS One. 2014 Nov 12;9(11):e110133. doi: 10.1371/journal.pone.0110133 (PMC4229085; doi:10.1371/journal.pone.0110133)
Supplement: Table S1 — ICD-9 procedure codes for surgical procedures. (DOCX) [file pone.0110133.s001.docx]

**Table S1: ICD-9 procedure codes for surgical procedures**

| **ICD-9 Code** | **Abbreviated procedure name** | **Study category** |
| --- | --- | --- |
| Cardiac Procedures | | |
| 3619 | Hrt revas byps anas nec | Cardiac_CABG |
| 3615 | 1 int mam-cor art bypass | Cardiac_CABG |
| 3611 | Aortocor bypas-1 cor art | Cardiac_CABG |
| 3610 | Aortocoronary bypass nos | Cardiac_CABG |
| 3614 | Aortcor bypas-4+ cor art | Cardiac_CABG |
| 3616 | 2 int mam-cor art bypass | Cardiac_CABG |
| 3617 | Abd-coron artery bypass | Cardiac_CABG |
| 3612 | Aortocor bypas-2 cor art | Cardiac_CABG |
| 3631 | Open chest trans revasc | Cardiac_CABG |
| 3632 | Oth transmyo revascular | Cardiac_CABG |
| 3633 | Endo transmyo revascular | Cardiac_CABG |
| 362 | Arterial implant revasc | Cardiac_CABG |
| 3634 | Perc transmyo revascular | Cardiac_CABG |
| 3639 | Oth heart revascular | Cardiac_CABG |
| 3613 | Aortocor bypas-3 cor art | Cardiac_CABG |
| 3609 | Rem of cor art obstr nec | Cardiac_PTCA/PCI |
| 0066 | Ptca or coronary ather | Cardiac_PTCA/PCI |
| 3601 | Ptca-1 ves/ath w/o agent | Cardiac_PTCA/PCI |
| 3602 | Ptca-1 ves/ath w agent | Cardiac_PTCA/PCI |
| 3603 | Open coronry angioplasty | Cardiac_PTCA/PCI |
| 3605 | Ptca-multiple vessel/ath | Cardiac_PTCA/PCI |
| 3606 | Ins nondrug elut cor st | Cardiac_PTCA/PCI |
| 3607 | Ins drug-elut coronry st | Cardiac_PTCA/PCI |
| 3606 | Ins nondrug elut cor st | Cardiac_PTCA/PCI |
| 3607 | Ins drug-elut coronry st | Cardiac_PTCA/PCI |
| 3503 | Closed pulmon valvotomy | Cardiac_Other |
| 3520 | Replace heart valve nos | Cardiac_Other |
| 3513 | Opn pulmon valvuloplasty | Cardiac_Other |
| 3512 | Opn mitral valvuloplasty | Cardiac_Other |
| 3500 | Closed valvotomy nos | Cardiac_Other |
| 3511 | Opn aortic valvuloplasty | Cardiac_Other |
| 3522 | Replace aortic valve nec | Cardiac_Other |
| 3504 | Closed tricusp valvotomy | Cardiac_Other |
| 3521 | Replace aort valv-tissue | Cardiac_Other |
| 3502 | Closed mitral valvotomy | Cardiac_Other |
| 3501 | Closed aortic valvotomy | Cardiac_Other |
| 3510 | Open valvuloplasty nos | Cardiac_Other |
| 3524 | Replace mitral valve nec | Cardiac_Other |
| 3525 | Replace pulm valv-tissue | Cardiac_Other |
| 3526 | Replace pulmon valve nec | Cardiac_Other |
| 3527 | Replace tric valv-tissue | Cardiac_Other |
| 3528 | Replace tricusp valv nec | Cardiac_Other |
| 3523 | Replace mitr valv-tissue | Cardiac_Other |
| 3514 | Opn tricus valvuloplasty | Cardiac_Other |
| 3963 | Cardioplegia | Cardiac_Other |
| 3963 | Cardioplegia | Cardiac_Other |
| 3961 | Extracorporeal circulat | Cardiac_Other |
| 3961 | Extracorporeal circulat | Cardiac_Other |
| 3962 | Hypothermia w open heart | Cardiac_Other |
| 3964 | Intraop cardiac pacemak | Cardiac_Other |
| 3965 | Extracorporeal memb oxy | Cardiac_Other |
| 3962 | Hypothermia w open heart | Cardiac_Other |
| 3964 | Intraop cardiac pacemak | Cardiac_Other |
| 3965 | Extracorporeal memb oxy | Cardiac_Other |
| 3966 | Per cardiopulmon bypass | Cardiac_Other |
| 3966 | Per cardiopulmon bypass | Cardiac_Other |
| 3844 | Resect abdm aorta w repl | Cardiac_Other |
| 3844 | Resect abdm aorta w repl | Cardiac_Other |
| 3971 | Endo impl grft abd aorta | Cardiac_Other |
| 3973 | Endo imp grft thor aorta | Cardiac_Other |
| 3834 | Aorta resection & anast | Cardiac_Other |
| 3560 | Grft repair hrt sept nos | Cardiac_Other |
| 3561 | Graft repair atrial def | Cardiac_Other |
| 3562 | Graft repair ventric def | Cardiac_Other |
| 3563 | Grft rep endocar cushion | Cardiac_Other |
| 3570 | Heart septa repair nos | Cardiac_Other |
| 3571 | Atria septa def rep nec | Cardiac_Other |
| 3572 | Ventr septa def rep nec | Cardiac_Other |
| 3573 | Endocar cushion rep nec | Cardiac_Other |
| 3581 | Tot repair tetral fallot | Cardiac_Other |
| 3760 | Imp bivn ext hrt ast sys | Cardiac_Other |
| 3583 | Tot rep truncus arterios | Cardiac_Other |
| 3755 | Rem int bivent hrt sys | Cardiac_Other |
| 3551 | Pros rep atrial def-opn | Cardiac_Other |
| 3598 | Other heart septa ops | Cardiac_Other |
| 3599 | Other heart valve ops | Cardiac_Other |
| 3531 | Papillary muscle ops | Cardiac_Other |
| 3533 | Annuloplasty | Cardiac_Other |
| 3535 | Trabecul carneae cord op | Cardiac_Other |
| 3539 | Tiss adj to valv ops nec | Cardiac_Other |
| 3541 | Enlarge existing sep def | Cardiac_Other |
| 3596 | Perc heart valvuloplasty | Cardiac_Other |
| 3550 | Prosth rep hrt septa nos | Cardiac_Other |
| 3532 | Chordae tendineae ops | Cardiac_Other |
| 3552 | Pros repair atria def-cl | Cardiac_Other |
| 3553 | Prost repair ventric def | Cardiac_Other |
| 3553 | Pros rep ventric def-opn | Cardiac_Other |
| 3554 | Pros rep endocar cushion | Cardiac_Other |
| 3555 | Pros rep ventrc def-clos | Cardiac_Other |
| 3754 | Repl/rep oth tot hrt sys | Cardiac_Other |
| 3591 | Interat ven retrn transp | Cardiac_Other |
| 3542 | Create septal defect | Cardiac_Other |
| 3582 | Total repair of tapvc | Cardiac_Other |
| 3753 | Repl/rep thorac unit hrt | Cardiac_Other |
| 3752 | Implant tot rep hrt sys | Cardiac_Other |
| 3751 | Heart transplantation | Cardiac_Other |
| 3735 | Partial ventriculectomy | Cardiac_Other |
| 3534 | Infundibulectomy | Cardiac_Other |
| 3732 | Heart aneurysm excision | Cardiac_Other |
| 3584 | Tot cor transpos grt ves | Cardiac_Other |
| 3699 | Heart vessel op nec | Cardiac_Other |
| 3691 | Coron vess aneurysm rep | Cardiac_Other |
| 3592 | Conduit rt vent-pul art | Cardiac_Other |
| 3733 | Exc/dest hrt lesion open | Cardiac_Other |
| 3754 | Repl/rep oth tot hrt sys | Cardiac_Other |
| 3599 | Other heart valve ops | Cardiac_Other |
| 3593 | Conduit left ventr-aorta | Cardiac_Other |
| 3594 | Conduit artium-pulm art | Cardiac_Other |
| 3595 | Heart repair revision | Cardiac_Other |
| 3596 | Perc heart valvuloplasty | Cardiac_Other |
| 3598 | Other heart septa ops | Cardiac_Other |
| GI Procedures | | |
| 4439 | Gastroenterostomy nec | GI_Gastric |
| 4467 | Lap creat esoph sphinct | GI_Gastric |
| 4498 | Adjust gast restrict dev | GI_Gastric |
| 4497 | Lap rem gast restric dev | GI_Gastric |
| 4496 | Lap rev gast restri proc | GI_Gastric |
| 4495 | Lap gastric restric proc | GI_Gastric |
| 4491 | Ligate gastric varices | GI_Gastric |
| 4469 | Gastric repair nec | GI_Gastric |
| 4468 | Laparoscop gastroplasty | GI_Gastric |
| 4499 | Gastric operation nec | GI_Gastric |
| 4399 | Total gastrectomy nec | GI_Gastric |
| 4311 | Percu endosc gastrostomy | GI_Gastric |
| 4319 | Other gastrostomy | GI_Gastric |
| 433 | Pyloromyotomy | GI_Gastric |
| 4341 | Endosc destr stomach les | GI_Gastric |
| 4342 | Local gastr excision nec | GI_Gastric |
| 4349 | Local gastr destruct nec | GI_Gastric |
| 435 | Proximal gastrectomy | GI_Gastric |
| 436 | Distal gastrectomy | GI_Gastric |
| 437 | Part gastrec w jej anast | GI_Gastric |
| 4381 | Part gast w jej transpos | GI_Gastric |
| 4441 | Sut gastric ulcer site | GI_Gastric |
| 4391 | Tot gast w intes interpo | GI_Gastric |
| 4466 | Creat esophagastr sphinc | GI_Gastric |
| 4421 | Dilate pylorus, incision | GI_Gastric |
| 4429 | Other pyloroplasty | GI_Gastric |
| 4431 | High gastric bypass | GI_Gastric |
| 4438 | Lap gastroenterostomy | GI_Gastric |
| 4440 | Suture peptic ulcer nos | GI_Gastric |
| 4442 | Suture duoden ulcer site | GI_Gastric |
| 445 | Revision gastric anastom | GI_Gastric |
| 4461 | Suture gastric lacerat | GI_Gastric |
| 4462 | Gastrostomy closure | GI_Gastric |
| 4463 | Close gastric fistul nec | GI_Gastric |
| 4464 | Gastropexy | GI_Gastric |
| 4465 | Esophagogastroplasty | GI_Gastric |
| 4389 | Partial gastrectomy nec | GI_Gastric |
| 5412 | Reopen recent lap site | GI_Laparotomies |
| 5451 | Lap periton adhesiolysis | GI_Laparotomies |
| 5411 | Exploratory laparotomy | GI_Laparotomies |
| 5419 | Laparotomy nec | GI_Laparotomies |
| 5459 | Oth periton adhesiolysis | GI_Laparotomies |
| 4258 | Thorac interposition nec | GI_Other |
| 4259 | Thorac esophag anast nec | GI_Other |
| 427 | Esophagomyotomy | GI_Other |
| 4269 | Stern esophag anast nec | GI_Other |
| 4268 | Stern interposition nec | GI_Other |
| 4266 | Stern esophagocolos nec | GI_Other |
| 4265 | Stern lg bowel interpos | GI_Other |
| 4264 | Stern esophagoenter nec | GI_Other |
| 4263 | Stern sm bowel interpos | GI_Other |
| 4255 | Thorac lg bowel interpos | GI_Other |
| 4261 | Stern esophagoesophagost | GI_Other |
| 4240 | Esophagectomy nos | GI_Other |
| 4256 | Thorac esophagocolos nec | GI_Other |
| 4254 | Thorac esophagoenter nec | GI_Other |
| 4253 | Thorac sm bowel interpos | GI_Other |
| 4252 | Thorac esophagogastrost | GI_Other |
| 4251 | Thorac esophagoesophagos | GI_Other |
| 4242 | Total esophagectomy | GI_Other |
| 4241 | Partial esophagectomy | GI_Other |
| 4262 | Stern esophagogastrostom | GI_Other |
| 4709 | Other appendectomy | GI_Other |
| 4701 | Lap appendectomy | GI_Other |
| 4719 | Other incid appendectomy | GI_Other |
| 472 | Drain appendiceal absc | GI_Other |
| 4791 | Appendicostomy | GI_Other |
| 4792 | Close appendiceal fistul | GI_Other |
| 4799 | Appendiceal ops nec | GI_Other |
| 4711 | Lap incid appendectomy | GI_Other |
| 4693 | Revise sm bowel anastom | GI_Other |
| 4681 | Intra-abd sm bowel manip | GI_Other |
| 4672 | Duodenal fistula closure | GI_Other |
| 4671 | Duodenal lacerat suture | GI_Other |
| 4602 | Resect ext seg sm bowel | GI_Other |
| 4601 | Sm bowel exteriorization | GI_Other |
| 4640 | Intest stoma revis nos | GI_Other |
| 4641 | Sm bowel stoma revision | GI_Other |
| 4673 | Small bowel suture nec | GI_Other |
| 4674 | Close sm bowel fist nec | GI_Other |
| 4651 | Sm bowel stoma closure | GI_Other |
| 4695 | Local sm bowel perfusion | GI_Other |
| 4660 | Intestinal fixation nos | GI_Other |
| 4661 | Sm bowel-abd wall fixat | GI_Other |
| 4662 | Small bowel fixation nec | GI_Other |
| 4650 | Intest stoma closure nos | GI_Other |
| 4642 | Pericolost hernia repair | GI_Other |
| 4643 | Lg bowel stoma revis nec | GI_Other |
| 4604 | Resect ext seg lg bowel | GI_Other |
| 4603 | Lg bowel exteriorization | GI_Other |
| 4571 | Opn mul seg lg intes nec | GI_Other |
| 4652 | Lg bowel stoma closure | GI_Other |
| 4679 | Repair of intestine nec | GI_Other |
| 4699 | Intestinal op nec | GI_Other |
| 4697 | Transplant of intestine | GI_Other |
| 4696 | Local lg bowel perfusion | GI_Other |
| 4694 | Revise lg bowel anastom | GI_Other |
| 4692 | Myotomy of colon nec | GI_Other |
| 4691 | Myotomy of sigmoid colon | GI_Other |
| 4685 | Dilation of intestine | GI_Other |
| 4574 | Transverse colon resect | GI_Other |
| 4680 | Intra-ab bowel manip nos | GI_Other |
| 4675 | Suture lg bowel lacerat | GI_Other |
| 4676 | Close lg bowel fistula | GI_Other |
| 4571 | Mult seg lg bowel excis | GI_Other |
| 4573 | Right hemicolectomy | GI_Other |
| 4575 | Left hemicolectomy | GI_Other |
| 4576 | Sigmoidectomy | GI_Other |
| 4579 | Part lg bowel excis nec | GI_Other |
| 458 | Tot intra-abd colectomy | GI_Other |
| 4682 | Intra-abd lg bowel manip | GI_Other |
| 4664 | Large bowel fixation nec | GI_Other |
| 4551 | Sm bowel segment isolat | GI_Other |
| 4500 | Intestinal incision nos | GI_Other |
| 4501 | Duodenal incision | GI_Other |
| 4502 | Small bowel incision nec | GI_Other |
| 4503 | Large bowel incision | GI_Other |
| 4531 | Local excision duodenum | GI_Other |
| 4531 | Oth excise duodenum les | GI_Other |
| 4532 | Destruct duoden les nec | GI_Other |
| 4533 | Local excis sm bowel nec | GI_Other |
| 4534 | Destr sm bowel les nec | GI_Other |
| 4595 | Anal anastomosis | GI_Other |
| 4550 | Intest seg isolat nos | GI_Other |
| 4663 | Lg bowel-abd wall fixat | GI_Other |
| 4552 | Lg bowel segment isolat | GI_Other |
| 4561 | Mult seg sm bowel excis | GI_Other |
| 4562 | Part sm bowel resect nec | GI_Other |
| 4563 | Total removal sm bowel | GI_Other |
| 4590 | Intestinal anastom nos | GI_Other |
| 4591 | Sm-to-sm bowel anastom | GI_Other |
| 4592 | Sm bowel-rect stump anas | GI_Other |
| 4593 | Small-to-large bowel nec | GI_Other |
| 4594 | Lg-to-lg bowel anastom | GI_Other |
| 4549 | Destruc lg bowel les nec | GI_Other |
| Orthopedic Procedures | | |
| 0072 | Rev hip repl-fem comp | Orthopedic_Hip |
| 0071 | Rev hip repl-acetab comp | Orthopedic_Hip |
| 0073 | Rev hip repl-liner/head | Orthopedic_Hip |
| 0074 | Hip repl surf-metal/poly | Orthopedic_Hip |
| 0074 | Hip surface, metal/poly | Orthopedic_Hip |
| 0075 | Hip rep surf-metal/metal | Orthopedic_Hip |
| 0075 | Hip surface, metal/metal | Orthopedic_Hip |
| 0076 | Hip rep surf-cermc/cermc | Orthopedic_Hip |
| 0076 | Hip surface, cermc/cermc | Orthopedic_Hip |
| 0077 | Hip repl surf-cermc/poly | Orthopedic_Hip |
| 0085 | Resrf hip,total-acet/fem | Orthopedic_Hip |
| 0086 | Resrf hip,part-fem head | Orthopedic_Hip |
| 8151 | Total hip replacement | Orthopedic_Hip |
| 8152 | Partial hip replacement | Orthopedic_Hip |
| 8153 | Revise hip replacement | Orthopedic_Hip |
| 8153 | Revise hip replacemt nos | Orthopedic_Hip |
| 0070 | Rev hip repl-acetab/fem | Orthopedic_Hip |
| 0087 | Resrf hip,part-acetablum | Orthopedic_Hip |
| 0081 | Rev knee repl-tibia comp | Orthopedic_Knee |
| 8142 | Five-in-one knee repair | Orthopedic_Knee |
| 8143 | Triad knee repair | Orthopedic_Knee |
| 8147 | Other repair of knee | Orthopedic_Knee |
| 8154 | Total knee replacement | Orthopedic_Knee |
| 8155 | Revise knee replacement | Orthopedic_Knee |
| 0080 | Rev knee replacemt-total | Orthopedic_Knee |
| 0082 | Rev knee repl-femur comp | Orthopedic_Knee |
| 0083 | Rev knee replace-patella | Orthopedic_Knee |
| 0084 | Rev knee repl-tibia lin | Orthopedic_Knee |
| 8155 | Revise knee replace nos | Orthopedic_Knee |
| 7855 | Internal fixation-femur | Orthopedic_Other |
| 7995 | Femur injury op nos | Orthopedic_Other |
| 7985 | Open reduc-hip dislocat | Orthopedic_Other |
| 7965 | Debrid opn fx-femur | Orthopedic_Other |
| 7915 | Closed red-int fix femur | Orthopedic_Other |
| 7905 | Cl fx reduc-femur | Orthopedic_Other |
| 7865 | Remove imp device-femur | Orthopedic_Other |
| 8181 | Partial shoulder replace | Orthopedic_Other |
| 8180 | Total shoulder replace | Orthopedic_Other |
| 8164 | Fus/refus 9 vertebrae | Orthopedic_Other |
| 8163 | Fus/refus 4-8 vertebrae | Orthopedic_Other |
| 8156 | Total ankle replacement | Orthopedic_Other |
| 8183 | Shoulder arthroplast nec | Orthopedic_Other |
| 7956 | Op red-tibia/fib epiphys | Orthopedic_Other |
| 7925 | Open reduction-femur fx | Orthopedic_Other |
| 7935 | Open reduc-int fix femur | Orthopedic_Other |
| 7920 | Open fx reduction nos | Orthopedic_Other |
| 7921 | Open reduc-humerus fx | Orthopedic_Other |
| 7926 | Open reduc-tibia/fib fx | Orthopedic_Other |
| 7931 | Open red-int fix humerus | Orthopedic_Other |
| 7935 | Open reduc-int fix femur | Orthopedic_Other |
| 7936 | Op red-int fix tib/fibul | Orthopedic_Other |
| 7950 | Open red-sep epiphy nos | Orthopedic_Other |
| 7955 | Opn red-sep epiphy-femur | Orthopedic_Other |
| 7959 | Open red-sep epiphy nec | Orthopedic_Other |
| 7960 | Open fx site debride nos | Orthopedic_Other |
| 7961 | Debrid open fx-humerus | Orthopedic_Other |
| 7925 | Open reduction-femur fx | Orthopedic_Other |
| 7951 | Opn red-sep epiphy-humer | Orthopedic_Other |
| 7955 | Opn red-sep epiphy-femur | Orthopedic_Other |
| 8004 | Arthrot/pros remov-hand | Orthopedic_Other |
| 8001 | Arthrot/pros remov-shldr | Orthopedic_Other |
| 8003 | Arthrot/pros remov-wrist | Orthopedic_Other |
| 8006 | Arthrot/pros remov-knee | Orthopedic_Other |
| 8007 | Arthrot/pros remov-ankle | Orthopedic_Other |
| 8008 | Arthrot/pros remov-foot | Orthopedic_Other |
| 8009 | Arthrot & pros remov nec | Orthopedic_Other |
| 8010 | Other arthrotomy nos | Orthopedic_Other |
| 8011 | Oth arthrotomy-shoulder | Orthopedic_Other |
| 8015 | Oth arthrotomy-hip | Orthopedic_Other |
| 8016 | Oth arthrotomy-knee | Orthopedic_Other |
| 8002 | Arthrot/pros remov-elbow | Orthopedic_Other |
| 8000 | Arthrot & pros remov nos | Orthopedic_Other |
| 8005 | Arthrot/pros remov-hip | Orthopedic_Other |
