# Supplementary material for: Postoperative Staphylococcus aureus Infections in Medicare Beneficiaries
Source: PLoS One. 2014 Nov 12;9(11):e110133. doi: 10.1371/journal.pone.0110133 (PMC4229085; doi:10.1371/journal.pone.0110133)
Supplement: Table S2 — ICD-9 diagnosis codes for infections. (DOCX) [file pone.0110133.s002.docx]

**Table S2: ICD-9 diagnosis codes for infections**

| **ICD-9 Code** | **Infection** | **Name of organism or subcategory** |
| --- | --- | --- |
| 0380 | Septicemia | Strep |
| 03810 | Septicemia* | Staph aureus unspecified |
| 03811 | Septicemia* | MSSA |
| 03819 | Septicemia* | MRSA |
| 0382 | Septicemia* | Staph |
| 0383 | Septicemia | Pneumo |
| 0384 | Septicemia | Gram neg |
| 03841 | Septicemia | H Flu |
| 03842 | Septicemia | E coli |
| 03843 | Septicemia | Pseudomonas |
| 03844 | Septicemia | Serratia |
| 03849 | Septicemia | Other |
| 0388 | Septicemia | Other Specified |
| 0389 | Septicemia | Other Non-specified |
| 041 | Infections of Unspecified Sites | Bacterial infection NOS |
| 0410 | Infections of Unspecified Sites | Strep |
| 0411 | Infections of Unspecified Sites* | Staph |
| 04111 | Infections of Unspecified Sites* | MSSA |
| 04112 | Infections of Unspecified Sites* | MRSA |
| 04119 | Infections of Unspecified Sites* | Other staph |
| 0412 | Infections of Unspecified Sites | Pneumo |
| 0413 | Infections of Unspecified Sites | Klebs Pniae |
| 0414 | Infections of Unspecified Sites | E coli |
| 0415 | Infections of Unspecified Sites | HI |
| 0416 | Infections of Unspecified Sites | Proteus |
| 0417 | Infections of Unspecified Sites | Pseudomonas |
| 0418 | Infections of Unspecified Sites | Other specified bacterial infections |
| 04181 | Infections of Unspecified Sites | Other |
| 04182 | Infections of Unspecified Sites | Other |
| 04183 | Infections of Unspecified Sites | Other |
| 04184 | Infections of Unspecified Sites | Other |
| 04185 | Infections of Unspecified Sites | Other |
| 04186 | Infections of Unspecified Sites | Other |
| 04189 | Infections of Unspecified Sites | Other |
| 0419 | Infections of Unspecified Sites | Not specified |
| 3200 | Bacterial Meningitis | H influenzae |
| 3201 | Bacterial Meningitis | Pneum |
| 3202 | Bacterial Meningitis | Strep |
| 3203 | Bacterial Meningitis* | Staph |
| 3207 | Bacterial Meningitis | NOS |
| 3208 | Bacterial Meningitis | Other Specified |
| 32081 | Bacterial Meningitis | Anerobic meningitis |
| 32082 | Bacterial Meningitis | Gram neg meningitis |
| 32089 | Bacterial Meningitis | Meningitis with other bacteria |
| 3209 | Bacterial Meningitis | Bacterium NOS |
| 4200 | Infections involving the Circulatory System | Acute pericarditis - diseases classified elsewhere |
| 42090 | Infections involving the Circulatory System | Acute pericarditis - NOS |
| 42091 | Infections involving the Circulatory System | Idiopathic pericarditis |
| 42099 | Infections involving the Circulatory System | Other pericarditis |
| 4210 | Infections involving the Circulatory System | Acute and subacute bacterial pericarditis |
| 4211 | Infections involving the Circulatory System | Acute and subacute bacterial pericarditis in diseases classified elsewhere |
| 4219 | Infections involving the Circulatory System | Acute endocarditis NOS |
| 4220 | Infections involving the Circulatory System | Acute myocarditis classified elsewhere |
| 4229 | Infections involving the Circulatory System | Other and unspecified myocarditis |
| 42290 | Infections involving the Circulatory System | Acute myocarditis not classified |
| 42291 | Infections involving the Circulatory System | Idiopathic myocarditis |
| 42292 | Infections involving the Circulatory System* | Septic myocarditis (Pneumo or Staph) - extra codes for organism e.g. staph 0411) |
| 42299 | Infections involving the Circulatory System | Other and unspecified myocarditis |
| 481 | Pneumonia | Pneumococcal pneumonia |
| 482 | Pneumonia | Other bacterial pneumonias |
| 4820 | Pneumonia | Klebsiella pnia |
| 4821 | Pneumonia | Pseudomonas |
| 4822 | Pneumonia | H flu |
| 4823 | Pneumonia | Strep |
| 48240 | Pneumonia* | Staph NOS |
| 48241 | Pneumonia* | MSSA |
| 48249 | Pneumonia* | Other staph aureus pneumonia |
| 4828 | Pneumonia | Other bacterial pneumonias |
| 4830 | Pneumonia | NA |
| 485 | Pneumonia | Bronchopneumonia NOS |
| 486 | Pneumonia | Pneumonia NOS |
| 5670 | Intestinal Infections | Peritonitis and retroperitoneal infections |
| 5671 | Intestinal Infections | Pneum peritonitis |
| 5672 | Intestinal Infections | Other supporative peritonitis |
| 5678 | Intestinal Infections | Other specified peritonitis |
| 5679 | Intestinal Infections | Unspecified peritonitis |
| 6820 | Infections of the Skin | Infection of face |
| 6822 | Infections of the Skin | Infection of trunk |
| 6826 | Infections of the Skin | Infection of the leg |
| 6829 | Infections of the Skin | Unspecified site |
| 70710 | Subcutaneous Infections | Ulcer lower limb, except pressure ulcer |
| 70711 | Subcutaneous Infections | Ulcer lower limb, except pressure ulcer |
| 7078 | Subcutaneous Infections | Chronic ulcer other sites |
| 7079 | Subcutaneous Infections | Chronic ulcer unspecified site |

* Denotes *S. aureus* infection.
