# Supplementary material for: Postoperative Staphylococcus aureus Infections in Medicare Beneficiaries
Source: PLoS One. 2014 Nov 12;9(11):e110133. doi: 10.1371/journal.pone.0110133 (PMC4229085; doi:10.1371/journal.pone.0110133)
Supplement: Table S3 — Adjusted predictors of S. aureus infection within 180 days following surgery by type of surgery based on odds ratios (OR). (DOCX) [file pone.0110133.s003.docx]

| Table S3: Adjusted predictors of *S. aureus* infection within 180 days following surgery by type of surgery based on odds ratios (OR) | | | | | | | | | | | | | | | | | | | | |
| --- | --- | --- | --- | --- | --- | --- | --- | --- | --- | --- | --- | --- | --- | --- | --- | --- | --- | --- | --- | --- |
|  | **Cardiovascular** | | | | |  | **Gastro-intestinal** | | | | |  | **Orthopedic** | | | | |  |  |  |
| **Predictor** | **CABG** | |  | **PCI** | |  | **Gastric** | |  | **Lapa-rotomy** | |  | **Hip** | |  | **Knee** | |  | **All** | |
| Female vs. male | 1.20 |  |  | 1.03 |  |  | 0.77 |  |  | 0.76 | * |  | 0.80 | * |  | 0.85 |  |  | 0.81 | * |
| Non-white vs. white | 0.71 |  |  | 0.98 |  |  | 1.11 |  |  | 1.04 |  |  | 1.21 |  |  | 0.99 |  |  | 1.08 |  |
| Age 75-84 vs. 00-74 yrs. | 1.06 |  |  | 1.05 |  |  | 0.93 |  |  | 0.88 |  |  | 1.08 |  |  | 1.04 |  |  | 1.01 |  |
| Age 85+ vs. 00-74 yrs. | 1.20 |  |  | 0.75 |  |  | 0.84 |  |  | 0.99 |  |  | 1.03 |  |  | 1.12 |  |  | 0.95 |  |
| Source: Acute or chronic hospitals or rehab vs. community | 1.04 |  |  | 1.28 | * |  | 1.64 | * |  | 1.37 |  |  | 1.24 |  |  | 3.61 | * |  | 1.43 | * |
| Disability and ESRD vs. age eligibility | 1.53 | * |  | 1.81 | * |  | 1.06 |  |  | 1.55 | * |  | 2.59 |  |  | 2.59 | * |  | 1.66 | * |
| Region MidWest vs. NEast and MidAtlan | 0.91 |  |  | 0.95 |  |  | 1.17 |  |  | 0.86 |  |  | 0.80 |  |  | 0.91 |  |  | 0.94 |  |
| Region South vs. NEast and MidAtlan | 0.93 |  |  | 0.87 |  |  | 1.24 | * |  | 1.01 |  |  | 1.20 |  |  | 0.99 |  |  | 1.05 |  |
| Region West vs NEast and MidAtlan | 1.02 |  |  | 0.93 |  |  | 1.59 | * |  | 0.90 |  |  | 1.20 |  |  | 1.22 |  |  | 1.18 | * |
| Elective vs. non-elective admission for index surgery | 0.92 |  |  | 0.70 | * |  | 0.64 | * |  | 1.16 |  |  | 0.60 | * |  | 0.48 | * |  | 0.76 | * |
| Diabetes (yes vs. no) | 1.62 | * |  | 1.35 | * |  | 1.18 | * |  | 1.24 |  |  | 1.28 | * |  | 1.37 | * |  | 1.29 | * |
| Congestive heart failure (yes vs. no) | 2.41 | * |  | 2.84 | * |  | 1.36 | * |  | 1.63 | * |  | 1.48 | * |  | 1.74 | * |  | 1.83 | * |
| Ischemic heart disease (yes vs. no) | n.a. |  |  | n.a. |  |  | 1.03 |  |  | 0.94 |  |  | 1.08 |  |  | 1.31 |  |  | 0.99 |  |
| COPD (yes vs. no) | 1.38 | * |  | 1.66 | * |  | 1.39 | * |  | 1.52 | * |  | 1.47 | * |  | 1.38 | * |  | 1.46 | * |
| Chronic renal disease (yes vs. no) | 2.39 | * |  | 3.01 | * |  | 1.25 | * |  | 2.06 | * |  | 1.70 | * |  | 2.32 | * |  | 1.91 | * |
| Solid cancer (yes vs. no) | 1.03 |  |  | 1.19 |  |  | 0.88 |  |  | 1.24 |  |  | 0.93 |  |  | 1.17 |  |  | 0.91 | * |
| Gastro-intestinal vs. cardiovascular surgery | n.a. |  |  | n.a. |  |  | n.a. |  |  | n.a. |  |  | n.a. |  |  | n.a. |  |  | 2.51 | * |
| Orthopedic vs. cardiovascular surgery | n.a. |  |  | n.a. |  |  | n.a. |  |  | n.a. |  |  | n.a. |  |  | n.a. |  |  | 3.12 | * |
| c-statistic | 0.73 |  |  | 0.78 |  |  | 0.64 |  |  | 0.69 |  |  | 0.70 |  |  | 0.72 |  |  | 0.73 |  |
| Note: *S. aureus* denotes *Staphylococcus aureus*. *S. aureus* infection refers to first hospitalization containing or following surgery of interest with discharge diagnoses including any ICD-9 code specific for infection due to *S. aureus*. Adjusted odds rates were obtained for each type of surgery and all surgeries combined by separate binomial logistic multivariate regressions, respectively. Rehab denotes rehabilitation; ESRD denotes end-stage renal disease; NEast denotes northeast; MidAtlan denotes mid-Atlantic; COPD denotes chronic obstructive pulmonary disease. | | | | | | | | | | | | | | | | | | | | |
